# The Pilot Study of the Influence of Free Ammonia on Membrane Fouling during the Partial Nitrosation of Pig Farm Anaerobic Digestion Liquid

**DOI:** 10.3390/membranes11110894

**Published:** 2021-11-19

**Authors:** Hanxiao Bian, Zhiping Zhu, Qianwen Sui, Shunli Wang

**Affiliations:** 1 Institute of Environment and Sustainable Development in Agriculture, Chinese Academy of Agricultural Sciences, Beijing 100081, China; bianhanxiao@126.com (H.B.); wshl6@126.com (S.W.); 2 Key Laboratory of Energy Conservation and Waste Management in Agricultural Structures, Ministry of Agriculture and Rural Affairs, Beijing 100081, China; 3 Research Center for Eco-Environmental Sciences, Chinese Academy of Sciences, Beijing 100085, China; qwsui@rcees.ac.cn; 4 Laboratory of Water Pollution Control Technology, Chinese Academy of Sciences, Beijing 100085, China

**Keywords:** membrane bioreactor, free ammonia, partial nitrosation, membrane fouling

## Abstract

The problem of membrane fouling is a key factor restricting the application of the membrane bioreactor (MBR) in the partial nitrosation (PN) and anaerobic ammonia oxidation (anammox) processes. In this study, the pilot-scale continuous flow MBR was used to start up the partial nitrosation process in order to investigate the change trend of mid-transmembrane pressure (TMP) in the process of start-up, which was further explored to clarify the membrane fouling mechanism in the pilot-scale reactor. The results showed that the MBR system was in a stable operating condition during the partial nitrosation operation and that the online automatic backwash operation mode is beneficial in alleviating membrane fouling and reducing the cost of membrane washing. Particular attention was paid to the influence trend of free ammonia (FA)on membrane fouling, and it was found that the increase in FA concentration plays the most critical role in membrane fouling. The increase in FA concentration led to an increase in the extracellular polymer (EPS), dissolved microorganism product (SMP) and soluble chemical oxygen demand (SCOD) concentration. FA was extremely significantly correlated with EPS and SCOD, and the FA concentration was approximately 20.7 mg/L. The SCOD_eff_ (effluent SCOD concentration) concentration was approximately 147 mg/L higher than the SCOD_inf_ (influent SCOD concentration) concentration. FA mainly affects membrane fouling by affecting the concentration of EPS and SCOD.

## 1. Introduction

Anaerobic ammonium oxidation (anammox) is an important nitrogen removal process and has many advantages, including no organic carbon consumption, less oxygen consumption and less sludge production, compared to the traditional nitrogen removal treatment [1]. Due to these benefits, it has been applied to the removal of the nitrogen of pig farm anaerobic digested liquid with high ammonia nitrogen (NH_4_^+^-N) and a low carbon–nitrogen ratio [2]. In the anammox process, anaerobic ammonia-oxidizing bacteria (AnAOB) directly convert nitrite (NO_2_^−^) and NH_4_^+^-N into nitrogen (N_2_) in a micro-oxygen environment [3,4]. Generally, partial nitrification (PN) is a front-end process for providing sufficient substrates for the anammox reaction, and its start-up and running stability are very important.

Some factors have been found to be able to influence the start-up of the PN process, including the hydraulic retention time (HRT), temperature, dissolved oxygen (DO), wastewater composition and nitrogen compound concentration [5,6]. Recently, the reactor configuration was also demonstrated to have a significant impact on the cultivation of bacteria in the PN and anammox process [7]. The membrane bioreactor (MBR) can effectively retain sludge and rapidly enrich targeted functional ammoxidation bacteria [8,9], which has been reported in an application of the PN and anammox process. Several studies have proved that the MBR exhibited an excellent performance for the PN and anammox process start-up [10,11,12]. Meanwhile, a continuous MBR has the advantages of a simple operation, easy control and stable operation [13,14], which is more conducive to large-scale sewage treatment. However, many problems (include membrane fouling) restrict the application of the MBR in the PN and anammox process [15,16]. Membrane fouling is mainly affected by the characteristics of sludge, membrane materials and the quality of treated water [17,18]. It has been found that membrane fouling of the MBR during the reactor operation period plays a crucial role in the instability and low efficiency of the PN and anammox process [12]. However, in the PN of the wastewater treatment process, few studies were conducted on the impact mechanisms of membrane pollution. Free ammonia (FA) is one of the key parameters impacting the PN and anammox processes [19], and a change in its concentration can impact the microbial community structure and properties of sludge. In particular, it can change the structure of the EPS and the components of the SMP [20]. Previous studies showed that the membrane fouling behavior is typically attributed to pore blocking and a cake formation [21], which was mainly caused by EPS and SMP [22,23]. Therefore, it is necessary to investigate the relationship between FA and membrane fouling parameters. To date, studies that investigated membrane fouling during the start-up of partial nitrification by using the MBR are very limited, and the impact of FA in the membrane fouling of MBR reactors for the partial nitrosation process is also rarely investigated.

Herein, the objective of this research is to analyze the PN-MBR process membrane fouling problems of pig farm anaerobic digestion liquid. The change trend of mid-transmembrane pressure (TMP) and the impact of the FA on the EPS, SMP and SCOD were further explored to clarify the membrane fouling mechanism in the pilot-scale reactor. This research provided certain technical support for the development of both membrane fouling control measures and engineering optimization in PN and anammox.

## 2. Material and Methods

### 2.1. Equipment of MBR Reactor

**Figure 1:**
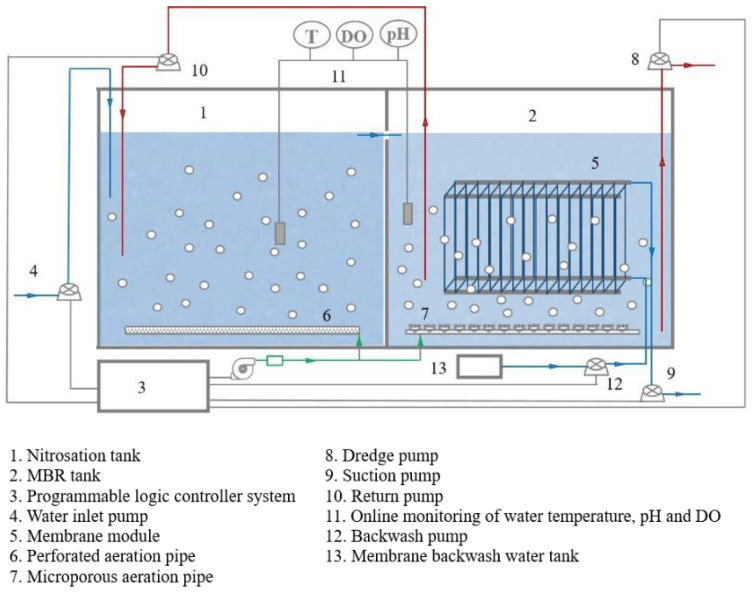
Schematic diagram of the pilot reactor device.

The pilot MBR reactor is shown in Figure 1. This reactor included two units: nitrosation tank and MBR tank. The total volume of nitrosation tank and MBR tank was 16 m³, and the effective volume was 12 m^3^. MBR membrane module was hollow fiber membrane (KH-MBR-8-co-PVDF, Hangzhou Kaihong Membrane Technology Co., Ltd., Hangzhou, China). There were microporous aeration pipes and temperature (T), DO and pH online monitors installed in the reactor. The sludge was well mixed in MBR tank and nitrification tank, and the remaining sludge was discharged through the sludge pump. In MBR tank, the membrane module was used to separate wastewaters into mixed sludge and water, and the drainage of water was completed by the suction pump. The reactor operation was automatically controlled by a programmable logic controller (PLC) system.

The MBR reactor setup and operation are as follows: the PLC system controlled the inlet water pump. The pig farm anaerobic digestion liquid entered the regulating tank for adjusting nitrogen of wastewaters, and then flowed into the nitrification tank and MBR tank. The HRT in the nitrification tank and MBR tank was 24 h, and the pH value was 8.0 ± 2.0. The two tanks were aerated intermittently. The aeration frequency in the start-up phase was 20 min:20 min (aeration: stop), and, in the stable operation, it was 30 min:10 min in order to keep DO at the level of 0.2~0.5 mg/L during aeration. The mixed sludge trapped in the MBR tank was returned to the nitrification tank with the return ratio of 200%. The solid retention time was controlled at 30 d. The water pumping of the MBR membrane module was set to 8 min on and 2 min off, with a suction cycle every 10 min. After 25 cycles were completed, the membrane module was backwashed automatically with clean water online, and the washing time was set to 10 min.

### 2.2. Influent and Inoculum Sludge

The pig farm anaerobic digestion liquid was collected from a pig farm in Hebei Province, China. After solid–liquid separation, the liquid part was used as the feedstock of this study. The activated sludge of PTA_2_O from sewage treatment plant was used as the inoculum. The characteristics of influent and inoculum sludge were shown in Table 1. A nitrogen source (NH_4_HCO_3_) was used to adjust ammonia nitrogen in the influent.

### 2.3. Online Membrane Wash

When transmembrane pressure difference in the membrane module reached approximately 40 kPa, the membrane module was cleaned by a washing system manually. The cleaning method was as follows:

(1) Cleaning agent (0.5% sodium hypochlorite) was placed in the clean water tank, and cleaning dosage was 2 L/m^2^ membrane surface area;
(2) Suction pump and filtering were stopped and the valve on the suction pump was closed;
(3) The aeration valve of the aeration blower and the membrane module were closed. After 1 min, the backwash pump was opened to inject the backwash liquid at a flow rate of 200 L/h. After injecting the backwash liquid for 10 min, the backwash pump was turned off. Then, after standing for 30 min, the backwash liquid was injected for another 10 min. After all of the backwash liquid was injected, the backwash pump was turned off. The valve on the pipeline was allowed to stand for 90 min;
(4) The membrane module aeration valve was opened, the aeration blower was turned on and aeration continued for 30 min;
(5) The aeration blower and aeration valve were closed, the valve on the suction pump pipeline was opened, the suction pump was opened and the liquid sodium hypochlorite in the membrane module was pumped out;
(6) If the effect of sodium hypochlorite washing was not obvious (indicated by high TMP), 1% citric acid was used to repeat the above steps to perform backwashing again.

### 2.4. Free Ammonia Impact Experiment on SCOD

A test of the influence of the different initial free ammonia concentrations on the SCOD concentration of effluent was conducted. In this experiment, the pH value was controlled at 8.0, the water temperature was controlled at 30 °C and different initial ammonia nitrogen concentrations were controlled. Different concentrations of free ammonia are calculated by a formula (Equation (1)). Specific operation steps are as follows: An appropriate amount of activated sludge was taken from MBR bank, and, after, the sludge with ammonia-free water was washed for three to four times. A total of 200 mL of the washed sludge was taken and placed in five 1000 mL beakers, and the prepared NH_4_Cl solution was added into each beaker to make the NH_4_^+^-N concentrations of 0 mg/L, 45.0 mg/L, 145 mg/L, 200 mg/L and 255 mg/L, respectively. The beaker was sealed with a sealing film and placed on a constant temperature magnetic stirrer (multi-head magnetic stirrer, HJ-6). The water temperature of the stirrer was controlled to 30 ± 1 °C, and the treatment was 24 h. The pH value of the sludge in each beaker was kept unchanged for 24 h, and, then, a small number of samples were taken from each beaker every 6 h to determine the MLSS, SCOD and NH_4_^+^-N of the treated samples.

### 2.5. Sampling and Analytical Methods

One hundred mL wastewater samples of influent and effluent of the MBR tank were taken at 9 am every day and stored in a refrigerator at 4 °C prior to the test. Mixed sludge samples were taken on day 1, 12, 13, 20, 30, 35, 39, 50 and 61 and stored in a freezer at −20 °C prior to the test.

Wastewater samples were filtered through 0.45 μm filter paper for MLSS, NH_4_^+^-N and SCOD analyses, which were conducted following the water and wastewater monitoring analysis method [24]. The TMP was monitored by the vacuum pressure gauge on the membrane module. The DO was detected using a portable DO detector, and water temperature was measured by an online thermometer. The calculation formula of FA is as follows [25].

(1) $$\mathrm{FA}=\frac{17}{14}\frac{\left[{\mathrm{NH}}_{4}^{+}-N\right]\times 10^{\mathrm{PH}}}{\exp\left(\frac{6334}{273+T}\right)+10^{\mathrm{PH}}}$$

where FA represents free ammonia concentration, mg/L^−1^; [NH_4_^+^-N] represents NH_4_^+^-N concentration, mg/L^−1^; T represents temperature, °C.

Mixed sludge samples of MBR tank were used to detect extracellular polymer (EPS) and soluble microbial products (SMP). For determination of SMP, the sludge sample was centrifuged at 4400× *g* for 30 min under 4 °C, the sludge was washed with 10 mL of ultrapure water and the washing liquid and the supernatant were combined to make a volume of 40 mL. For determination of EPS, the sludge was washed and suspended in 20 mL of ultrapure water, added with 120 μL of formaldehyde, placed at 4 °C for 1 h and then added with 8 mL of 1 moL/L NaOH solution. Then, the solution was placed at 4 °C for 3 h and centrifuged at 10,000× *g* for 20 min, and the supernatant set the volume to 40 mL. The protein and polysaccharide in EPS and SMP could be determined by a phenol–sulfuric acid method [26] and Lowry method [27].

## 3. Results and Discussion

### 3.1. Reactor Operation Effect and Sludge Characteristics

The equipment operated stably for 73 days; when the NH_4_^+^-N concentration in the influent was 400 mg/L, the ammonia oxidation rate was 50% and the NO_2_^−^-N and NH_4_^+^-N concentrations in the effluent were 198 ± 27.5 mg/L and 215 ± 33.9 mg/L, respectively. NO_2_^−^-N/NH_4_^+^-N was stable at 1.1:1, and the accumulation rate of NO_2_^−^-N was stable at a high level, with the highest value of 88.0% [28]. Previous studies have shown that the SHARON-anammox requires the ratio of the effluent NO_2_^−^-N concentration to NH_4_^+^-N concentration in PN to be between 1.3:1.0 [29]. In addition, simultaneous nitrification–denitrification and single-stage PN-anammox requires a high concentration of NO_2_^−^-N as the reaction matrix [30,31]. The results of this experiment provide a research foundation for the subsequent denitrification process.

### 3.2. Transmembrane Pressure Changes

**Figure 2:**
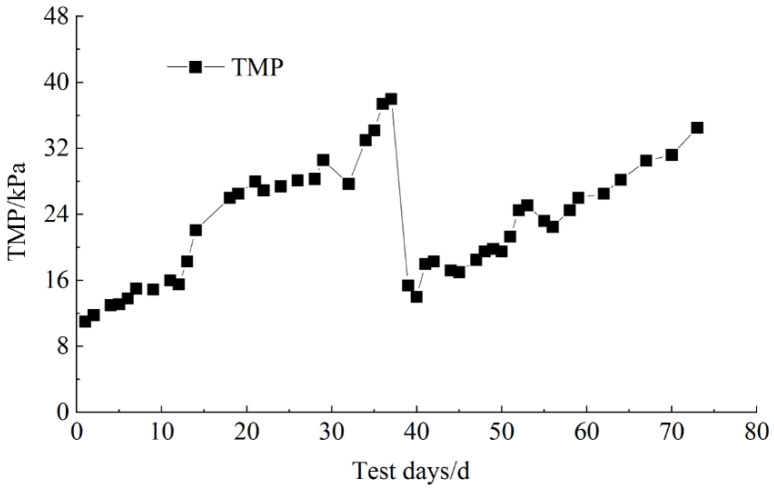
Changes in transmembrane pressure during partial nitrosation process.

The direct characterization of membrane fouling was the change in the TMP, and the changing trend of TMP is shown in Figure 2. In the initial days, the membrane flux was maintained at 13.3 L/(m^2^·h), and the TMP increased gradually from 11.0 kPa to 15.5 kPa. On the 14th day, the membrane flux dropped to approximately 11.3 L/(m^2^·h), and the initial membrane flux was maintained by adjusting the effluent flow, which resulted in the TMP increasing to 22 kPa. The reactor was operated on for 37 days, the TMP was increased to 38 kPa and the membrane flux was significantly reduced. A total of 0.5% sodium hypochlorite solution and 1% critic acid solution were used to clean the membrane module in sequence. After cleaning using the sodium chlorate solution, the TMP was reduced to 23 kPa, and, after cleaning using the citric acid solution, the TMP was reduced to 14 kPa. The phenomenon of the online chemical washing not restoring the original value of the TMP of the membrane module was caused by the blockage of the membrane pores by small particles of sludge [9]. When the reactor was operated to the 73rd day, the TMP difference in the membrane module increased to 34 kPa, and the MBR system operated stably. During the treatment of livestock wastewater, the membrane fouling rate of the MBR reactor was relatively fast [32]. The experiment used the intermittent backwashing of the membrane effluent to reach 30 kPa on the 29th day. Previous research has shown that synchronous aeration and water backwashing contributes to the recovery of the permeate flux [33]. Compared with the running process without backwashing [32], this study slowed down the rate of membrane fouling to a certain extent, and reduced the cost of chemical washing, which provides a reference for the practical application of membrane bioreactors.

### 3.3. Extracellular Polymer (EPS) and Dissolved Microorganism Product (SMP) Changes

**Figure 3:**
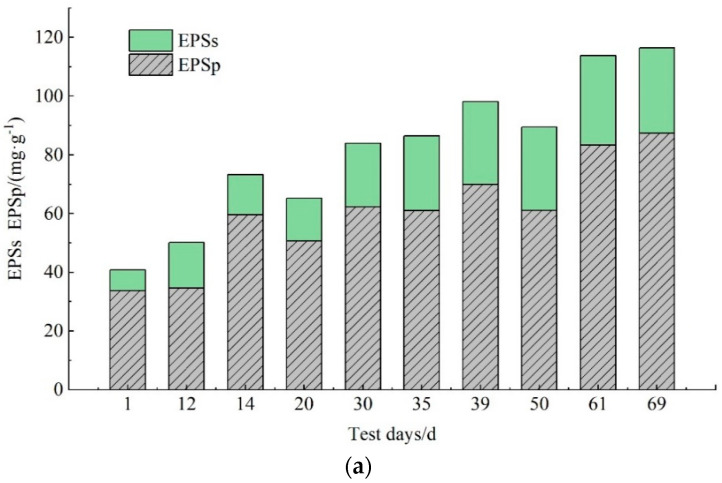

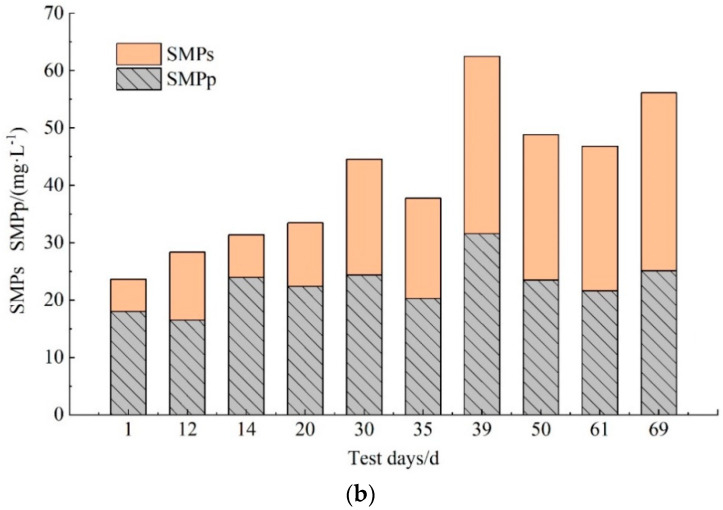
Changes in the content of polysaccharides and protein in (**a**) EPS and (**b**) SMP under different FA. EPSs, SMP_S_ are polysaccharides and EPS_P_, SMP_P_ are proteins, respectively.

**Figure 4:**
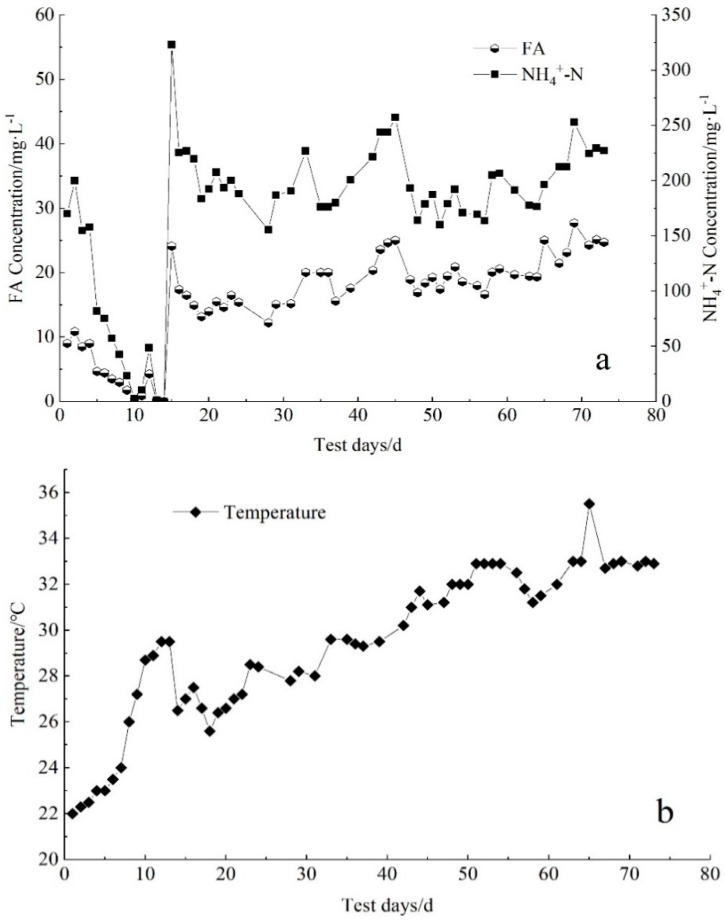
Changes in (**a**) FA and NH_4_^+^-N concentration and (**b**) temperature during partial nitrosation process.

EPS and SMP are the important indicators that characterize membrane fouling [12,34]. EPS adheres to the surface of the membrane module, causing an increase in TMP, and gradually decreasing the stability of the water output. The stable operation of the sewage system was affected, and the application of membrane bioreactors in practical engineering was limited [35]. Polysaccharides and proteins are the main components in SMP and EPS; the changes in their content in EPS and SMP are shown in Figure 3. FA is a by-product of the degradation of nitrogen-containing organic matter, and its concentration depends on the concentration of NH_4_^+^-N, pH and temperature [36]. The changes in FA, NH_4_^+^-N concentration and temperature are shown in Figure 4.

With the increase in FA concentration, the content of the EPS and SMP proteins and polysaccharides increased. As shown in Figure 3a, the EPS content showed an upward trend in the range of 40.8 mg/g~116 mg/g. The content of EPS_P_ was always higher than that of EPS_S_, and EPS_P_ may have a very important positive correlation with membrane fouling. The EPS content in the initial mixed sludge was as low as 41.0 mg/g, and the EPS_S_ and EPS_P_ contents were 7.05 mg/g and 33.8 mg/g, respectively. On the 12th day, the EPS_S_ content was 15.5 mg/g and the EPS_P_ content was 34.7 mg/g. The increase in NH_4_^+^-N loads led to an increase in the FA concentration to 24.1 mg/L. The EPS_S_ content declined slightly to 14.0 mg/g, and the EPS_P_ content was significantly increased to 59.6 mg/g. From day 22 to day 69, as the temperature gradually rose, the FA concentration increased from 13.6 mg/L to 27.7 mg/L, and the EPS content increased to 116 mg/L.

As shown in Figure 3b, before the chemical cleaning of membrane modules, the SMP concentration range was 23.7 mg/L~37.7 mg/L. The SMP_P_ content was high, based on the content of SMP_S_ in this stage, and the contents of SMP_P_ and SMP_S_ were 18.0 mg/L and 5.65 mg/L, respectively. The process ran to the 35th day, and the SMP concentration was raised to 37.8 mg/L (which includes SMP_P_ of 20.3 mg/L and SMP_S_ of 17.5 mg/L). Then, on day 37, the membrane module was chemically cleaned and the SMP increased to the highest concentration, which was 62.0 mg/L. The change in SMP concentration was related to the NaClO added during chemical cleaning. The residual NaClO solution from the cleaning diffused into the sludge mixture and had a destructive effect on the sludge flocs, and more intracellular substances were released into the sludge [37,38]. From day 39 to day 61, the SMP decreased to 46.8 mg/L, because SMP is a metabolically active carbon source and, after the membrane cleaning, can be gradually degraded by microorganisms in the sludge mixture. From day 50 to day 69, the SMP_P_ content was lower than the SMP_S_ content. On day 69, the SMP content increased to 56.2 mg/L, and the SMP_P_ and SMP_S_ contents were 25.2 mg/L and 31.0 mg/L, respectively.

FA has been reported to break down the EPS and damage the living cells [20], but some research shows that, with an increase in initial free ammonia concentrations from 0.50 to 10.0 mg/L, the production of the EPS component significantly increased [39]. This research proved that, in the actual operation process, when the FA concentration changed from 4.27 to 27.7 mg/L, the increase in the FA concentration will lead to an increase in the EPS and SMP concentration.

### 3.4. Changes in SCOD

**Figure 5:**
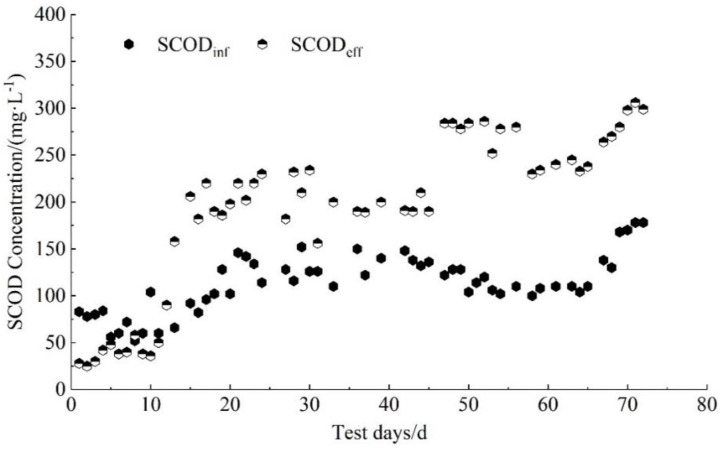
Changes in SCOD during the initiation of partial nitrosation process.

SCOD represents the soluble nutrient matrix in the reactor [40], which has a significant correlation with EPS. In a submerged MBR, SCOD concentration is a key factor affecting membrane fouling [41]. Therefore, the SCOD changes during the experiment were monitored, as shown in Figure 5. At the initial stage, the SCOD_inf_ concentration was slightly higher than the SCOD_eff_ concentration by approximately 25.0 mg/L, which was possibly caused by carbon consumption in the microbial growth in the reactor. The FA concentration was still at a low level, at an average concentration of 5.00 mg/L. As the experiment proceeded, the SCOD_eff_ concentration was gradually higher than the SCOD_inf_ concentration. It was found that, when the FA concentration was around 16.0 mg/L, the SCOD_eff_ concentration was approximately 85.0 mg/L higher than the SCOD_inf_ concentration on average. At the end of the experiment, when the FA concentration was approximately 21.0 mg/L, the SCOD_eff_ concentration was approximately 147 mg/L, higher than the SCOD_inf_ concentration. The results show that, within a certain range, an increase in FA concentration could promote the release of more SCOD in the reactor and provide more biodegradable organic substrates. The diversity of microorganisms gradually increased, the microbiota became increasingly active and the micro-ecological structure became more complex, as shown in Table 2 [28].

**Figure 6:**
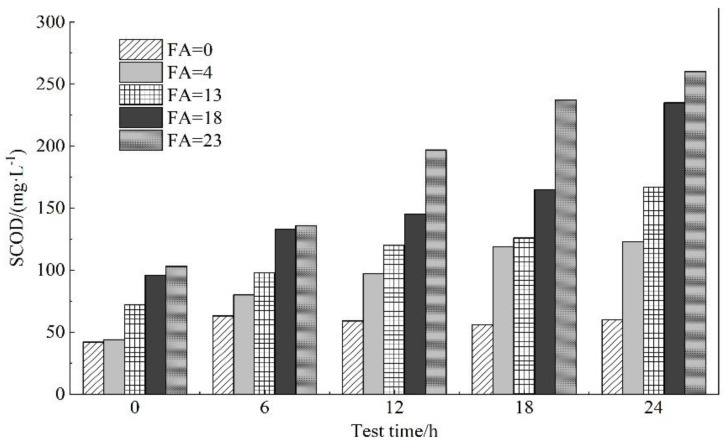
The influence of different FA on SCOD in sludge.

Figure 6 showed that different initial FA concentrations have an influence on the SCOD concentration. Compared with the blank group, with the gradual increase in FA, the concentration of SCOD in the effluent also gradually increased. Within 24 h, when the FA concentration was 13.0 mg/L, compared with the FA concentration of 0 mg/L, the SCOD content was higher than the blank group by 107 mg/L. When the FA concentration was 18.0 mg/L, compared with the FA concentration of 0 mg/L, the SCOD content was higher than the blank group by 175 mg/L, which was similar to the pilot test results. A higher concentration of FA will cause the dissolution of SCOD in the reactor and destroy the structure of the sludge, which will have a negative impact on membrane fouling.

### 3.5. The Relationship between FA and EPS, SMP, SCOD, TMP

The Pearson’s correlation coefficients between FA and EPS, SMP, SCOD and TMP were analyzed to reflect the relationship between the various parameters. As can be seen from Table 3, there was a strong correlation between EPS, SMP, SCOD and TMP, and it can be concluded that membrane fouling was the result of the interaction of multiple parameters. This is similar to the results of Wang ZW et al., which were that the membrane fouling is not impacted by a single factor of certain sludge properties, but a relatively complex result of the combined use of multiple properties of sludge [42]. In addition, the EPS, SMP, SCOD and TMP Pearson’s correlation coefficients (r_p_) were 0.780, 0.600, 0.797 and 0.538, respectively, which means that FA is extremely significantly correlated with EPS and SCOD, but has no significant correlation with SMP and TMP. From these results, it can be seen that FA mainly affects membrane fouling by affecting the concentration of EPS and SCOD.

## 4. Conclusions

This study found that the online automatic backwash operation mode was conducive to alleviating membrane pollution and reducing the cost of membrane washing during the partial nitrosation of pig farm anaerobic digestion liquid. TMP showed an upward trend during the whole start-up process, and it increased to 38 kPa after the experiment was run for 37 days. The MBR system operates stably in the actual operation process. The FA concentration was one of the influencing factors of membrane fouling and was extremely significantly correlated with EPS and SCOD, and, when the FA concentration was approximately 20.7 mg/L, the SCOD_eff_ concentration was approximately 147 mg/L higher than the SCOD_inf_ concentration. The FA mainly affects membrane fouling by affecting the concentration of EPS and SCOD.

## Figures and Tables

**Table 1:** The influent and initial sludge characteristics.

NH_4_^+^-N/mg·L^−1^	SCOD ^a^/mg·L^−1^	MLSS ^b^/g·L^−1^	MLVSS ^c^/g·L^−1^	SV30 ^d^/%	pH
153–508	52.0–178	4.73	3.49	38	8.0

^a^ Soluble chemical oxygen demand. ^b^ Mixed liquor suspended solids. ^c^ Mixed liquid volatile suspended solids. ^d^ The volume percentage of sludge after the sludge mixture settles for 30 min.

**Table 2:** Microbial community richness and diversity index.

Test Days/d	Coverage	Chao	Simpson	Ace
1	0.989	2559.273	0.018	2585.432
39	0.986	3738.099	0.009	3819.655

**Table 3:** Correlation between parameters.

Analysis Project	EPS		SMP		SCOD		TMP		FA	
	r_p_	*p*	r_p_	*p*	r_p_	*p*	r_p_	*p*	r_p_	*p*
EPS	1		0.863 **	0.001	0.847 *	0.002	0.967 **	0.000	0.780 **	0.008
SMP	0.863 **	0.001	1		0.730 *	0.017	0.856 *	0.014	0.600	0.067
SCOD	0.847 *	0.002	0.730 *	0.017	1		0.827 *	0.022	0.797 **	0.006
TMP	0.967 **	0.000	0.856 *	0.014	0.827 *	0.022	1		0.538	0.213
FA	0.780 **	0.008	0.600	0.067	0.797 **	0.006	0.538	0.213	1	

Note: * represents that the degree of significance is at the 0.05 level (*p* < 0.05); ** represents the significance level at 0.01 level (*p* < 0.01).

## Data Availability

The data presented in this study are available on request from the corresponding author.
